# Cystic burden meets blood pressure: cardiac remodelling in paediatric autosomal dominant polycystic kidney disease

**DOI:** 10.1007/s00467-026-07312-8

**Published:** 2026-05-18

**Authors:** Camille Heming, Emily Haseler, Alexandra Savis, Hannah Samuel, John M. Simpson, Manish D. Sinha

**Affiliations:** 1 Department of Paediatric Nephrology, 3rd Floor Becket House, Evelina London Children’s Hospital, Guys & St Thomas’ NHS Foundation Trust, Westminster Bridge Road, London, SE1 7EH UK; 2 Department of Clinical Pharmacology, School of Cardiovascular and Metabolic Medicine, King’s College London, London, UK; 3 Department of Paediatric Cardiology, Evelina London Children’s Hospital, Guys & St Thomas’ NHS Foundation Trust, Westminster Bridge Road, London, SE1 7EH UK

**Keywords:** Hypertension, Cyst burden, Cardiac remodelling, ADPKD

## Abstract

**Background:**

We evaluated the prevalence and phenotype of hypertension in untreated children with autosomal dominant polycystic kidney disease (ADPKD) and examined associations with blood pressure (BP), cyst burden, and left ventricular (LV) structure and function.

**Methods:**

Single-centre, cross-sectional study included children and young people (< 18 years) with ADPKD. Data regarding demographics, office BP,  24-h ambulatory BP monitoring if > 5-years, biochemistry and echocardiography were collated. Cyst burden was assessed by kidney ultrasound and classified as ‘low burden’ or ‘high burden’. Participants with hypertension (BP ≥ 95th percentile) or ‘high-normal’ BP (BP ≥ 90th and < 95th percentile) were grouped as ‘High BP’ (BP ≥ 90th percentile), and the remaining as normotensive (BP < 90th percentile).

**Results:**

Amongst 116 children (mean age 9.99 ± 5.2 years; 57.8% female), hypertension prevalence was 12% (*n* = 14), 8 with masked hypertension. Those with High BP (26% (*n* = 30)) had a higher cyst burden (56% vs. 34%, *P* = 0.036), LV hypertrophy (LVH) (16.6% vs. 4.6%, *P* = 0.034) and relative LV wall thickness (0.34 ± 0.08 vs. 0.29 ± 0.05, *P* < 0.001) but similar indexed LV mass (LVMI) and LV function when compared with normotensives. In multivariable analysis, cyst burden independently predicted LVMI (standardised *β* = 0.23, *P* = 0.02), whereas systolic BP did not (standardised β = 0.19, *P* = 0.14).

**Conclusions:**

Hypertension affected 12% of untreated children with ADPKD and preserved kidney function. High BP was associated with increased prevalence of LVH and relative wall thickness when compared to those who were normotensive. Cyst burden correlated with adverse cardiac remodelling, suggesting a BP-independent mechanism.

**Graphical Abstract:**

**Figure:**
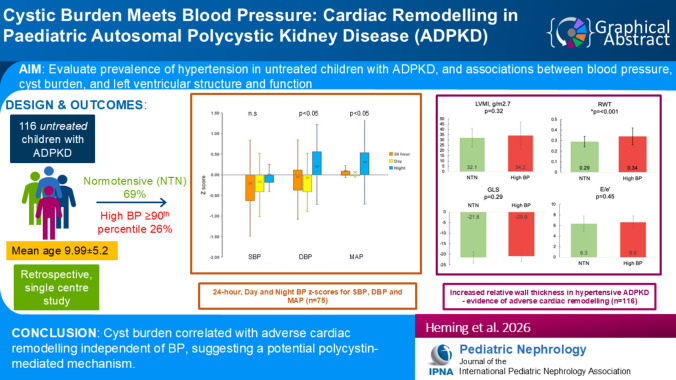
A higher resolution version of the Graphical abstract is available as Supplementary information

**Supplementary Information:**

The online version contains supplementary material available at 10.1007/s00467-026-07312-8.

## Introduction

Adults with autosomal dominant polycystic kidney disease (ADPKD) comprise nearly 10% of all patients with kidney disease receiving dialysis or kidney transplantation in Europe [1, 2]. ADPKD remains the most common inherited kidney disease, with progressive loss of kidney function resulting in need for kidney replacement therapy in 50% of those affected by their 6th decade [2, 3]. Mutations in the *PKD1* gene coding for the protein polycystin 1 (PC1) are most common, followed by those in the *PKD2* gene coding for the protein polycystin 2 (PC2) [4]. Most children with ADPKD presenting with cystic kidney involvement have *PKD1* or *PKD2* gene mutations with other less common gene mutations in ADPKD seen mostly presenting in adulthood [1, 3, 4]. Dysfunction in ciliary proteins PC1 and PC2 results in abnormal proliferation of the kidney tubular epithelium and kidney cyst formation leading to progressive loss of kidney function, but also results in extrarenal involvement as polycystin is expressed in other organs, such as the liver and vascular smooth muscle cells [2–4].

Hypertensive adults with ADPKD have evidence of hypertension mediated organ damage (HMOD) and increased cardiovascular morbidity and mortality [5]. A systematic review and meta-analysis evaluating the prevalence of hypertension in ~ 1000 affected children with ADPKD estimated hypertension in nearly 20%, with the highest prevalence in those with increased cyst burden and older adolescents [6]. Most hypertensive children with ADPKD are asymptomatic and have normal kidney function, however markers of cardiovascular morbidity are described in few studies, mostly with limited participants and including those receiving anti-hypertensive therapy [7–11]. The precise aetiology of hypertension in ADPKD is unclear and thought to be multifactorial while a principal contributory role is attributed to  the renin–angiotensin–aldosterone system (RAAS) - activated secondary to local perfusion abnormalities secondary to cystic involvement - the sympathetic nervous system and endothelial dysfunction have also been implicated [7, 12–14].

In keeping with our previous findings highlighting the relevance of blood pressure (BP) in this population [6, 7], we hypothesised that elevated BP is the major determinant of adverse cardiac remodelling in untreated children with ADPKD. Our aim in this report is to (i) evaluate the prevalence, severity and phenotype of hypertension and High BP; and (ii) to investigate the relationship of BP with cyst burden and LV structure and function in untreated children with a clinical diagnosis of ADPKD.

## Methods

This is a single-centre, retrospective, cross-sectional study of all consecutive children referred to a dedicated tertiary paediatric ADPKD clinic. We included children and young people (aged < 18 years) with a clinical diagnosis of ADPKD. Individuals were deemed to have ADPKD if (i) confirmed through ultrasonography using the Modified Ravine criteria [15] or (ii) in those aged under 15 years, a finding of kidney cysts in the context of a family history of ADPKD – criteria used in the recent large pan-European study [12] or (iii) confirmation of ADPKD following genetic testing.

### Inclusion and exclusion criteria

All included had: (i) suitable echocardiogram study; (ii) standardised auscultatory office BP measurement in all and a 24-h ambulatory blood pressure monitoring (ABPM) study where tolerated in those > 5-years; and (iii) not receiving anti-hypertensive medication. The patient was excluded if any of the following were present: (i) cystic kidney disease secondary to other known cause; (ii) unilateral cysts in the absence of a family history of ADPKD; (iii) those with any congenital or structural heart disease; (iv) those unable to tolerate office BP assessment or echocardiography. This was a retrospective analysis evaluating results of clinical investigations performed previously as part of routine clinical care. As per our institutional guidelines no consent from patients was indicated and ethical approval was not required. Use of echocardiographic data on imaging archive for this study was approved by the local research ethics committee (Research Ethics Committee reference: 09/H0802/116*).*

We collected baseline data including demographics, latest kidney ultrasound findings and relevant clinical and family history. Data for office BP, 24-h ABPM, echocardiography (LV structure and function), and relevant biochemical characteristics were also collected. Estimated glomerular filtration rate (eGFR) was calculated using the new Modified Schwartz formula from 2009 [16, 17]. UK childhood population reference values for height and weight were used to calculate z-scores [18, 19].

### Measurement of blood pressure and definition of hypertension

Standardised seated auscultatory office BP was performed following 5 min of rest, using a validated aneroid sphygmomanometer, and average of three consecutive BP measurements taken. We performed ABPM monitoring on all children older than 5-years. During ABPM, measurements were performed every 30 min through the 24-h period.

In those < 16 years old, we defined hypertension if the average ABPM result for systolic, mean or diastolic blood pressure was ≥ 95th percentile in any of the 24-h, awake or sleep periods. ‘High-normal BP’ was defined as ≥ 90th and < 95th percentile and 'normal BP' as < 90th percentile.

For those 16 years or older we defined hypertension using adult cut-off values rather than percentiles (24-h average ≥ 130/80, Daytime ≥ 135/85 and Night-time ≥ 125/75 mmHg [20].

If ABPM was not available, office BP was used to define hypertension. In those < 16 years old, we defined hypertension as ≥ 95th percentile for age, sex, and height. ‘High-normal BP’ was defined as ≥ 90th and < 95th percentile and normal BP as < 90th percentile. For those 16 years or older we defined hypertension using office BP as ≥ 140/90, and high-normal BP as 130–139/95–89 [20, 21].

To evaluate the relevance of elevated BP in this high-risk population of childhood onset of chronic kidney disease (CKD), we evaluated the ‘hypertension’ and ‘high-normal’ patients in a single category ‘High BP’ group.

In keeping with the recent KDIGO-ADPKD guidelines, we also evaluated our study findings in those with BP levels equal to or above and below the 75th percentile [21, 22].

Sustained hypertension was defined if hypertensive on both clinic BP and ABPM. Masked hypertension was defined if normotensive on clinic BP and hypertensive on ABPM. White coat hypertension (WCH) was defined if hypertensive on clinic BP but normotensive on ABPM. The absence of nocturnal dipping (‘non-dipping’) was defined as a nocturnal BP fall of < 10% daytime values [12].

### Echocardiographic assessments

All echocardiography studies were performed as part of routine clinical care and most on the same day as the ABPM study. All studies were interpreted by one experienced paediatric cardiac physiologist, AS. Left ventricular structural, geometrical and functional parameters were assessed using two-dimensional transthoracic echocardiography. Left ventricular hypertrophy (LVH) was defined if ≥ 95th percentile using age-specific reference interval indexed LV mass (LVMI in g/m^2.7^) [23–25]. Abnormal LV geometry was categorised as concentric remodelling (normal LVMI and increased relative wall thickness [RWT] > 0.41), eccentric LVH (increased LVMI ≥ 95th percentile age and gender, normal RWT) or concentric LVH (increased LVMI ≥ 95th percentile age and gender, increased RWT > 0.41) [26]. Echocardiography measurements were carried out in accordance with established guidelines (American Society of Echocardiography recommendations) [27]. To be consistent with previous reports considering the relationship of LV mass with BP in childhood ADPKD, we additionally reported LVMI in g/m^2^ [8].

### ADPKD disease severity

**Fig. 1:**
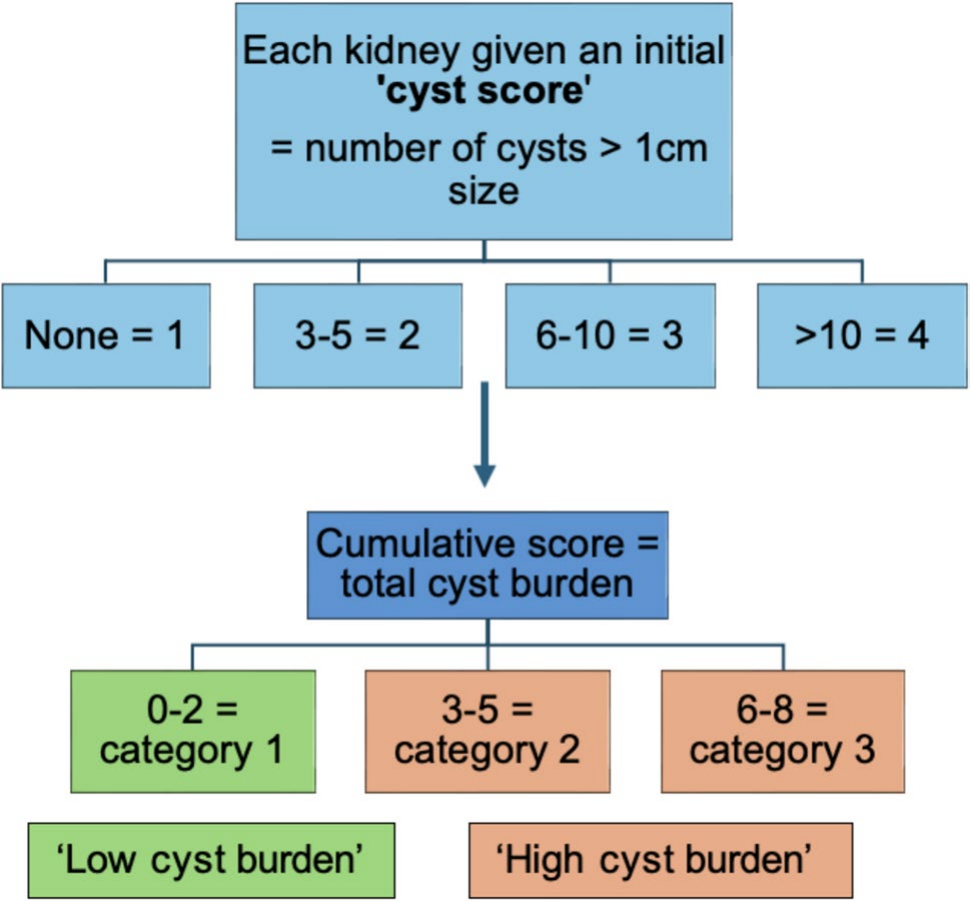
Evaluation of cyst burden to ‘low burden’ and ‘high burden’ categories [12]. * ‘cyst score’ calculated per kidney and added to give overall cyst burden

Cyst burden was assessed using kidney ultrasound, based on number of cysts above 1 cm diameter present in each kidney, as per recently published classification to maintain continuity in literature [12]. Subsequent analysis categorised cyst burden for both kidneys into two groups, ‘*low burden*’ (cyst burden score 0–2) or ‘*high burden*’ (cyst burden score ≥ 3) (Fig. 1).

### Statistical analysis

All data was analysed using IBM SPSS Statistics for Macintosh, Version 31.0 (IBM Corp, Armonk, NY). Averages were reported as means ± standard deviation (SD) if normally distributed, and median (interquartile range) if non-normally distributed. Categorical variables were compared using Chi squared test and continuous variables using independent t test with Bonferroni post hoc analysis for multiple comparisons. Where data were not normally distributed, the non-parametric Mann–Whitney U test was used for independent samples, and P < 0.05 was used to denote statistical significance.

A multiple linear regression analysis model was used to explore the relationship between LVMI (g/m^2.7^) as a dependent variable, with independent variables including cyst burden score (low or high), office systolic blood pressure (SBP), sex, age, eGFR and body mass index (BMI) z-score. An alternative model with BP level as a dependent variable and the same confounders but also including LVMI was additionally examined. Standardised β-coefficients were used to assess strength of each variable’s relationship with LVMI, adjusted for relevant co-variants. The model’s goodness-of-fit was expressed using adjusted r^2^, and multicollinearity was quantified using the variance inflation factor (upper acceptable limit = 4). P-value < 0.05 was considered statistically significant, and all tests were 2-tailed.

## Results

We included 116 children presenting consecutively with ADPKD to our clinic with mean age 9.99 ± 5.2, of whom 57.8% were girls and 62.1% were of White ethnicity.

We evaluated electronic health records of n = 122 consecutive children with a clinical diagnosis of ADPKD who had both blood pressure and echocardiography performed. We excluded six children (four were on anti-hypertensive medication, and two as there were no office BP measurements available). Echocardiography was performed successfully in all children.

### Prevalence of hypertension and its phenotype in untreated children with ADPKD

Seventy-five children successfully tolerated  both ABPM and standardised office BP measurements. Of the 41 children with office BP measurements alone and no ABPM, 53% (n = 22/41) were < 5 years old and 46% (n = 19/41) unable to tolerate ABPM. All included children had office SBP and n = 14 had missing diastolic BP (DBP) measurements.

The prevalence of hypertension was 12% (n = 14/116) of whom 8 children had masked hypertension. Table 1 (a) and (b) show the prevalence of hypertension and phenotypes using 24-h ABPM or standardised office BP measurement. Table 1 (c) shows hypertension prevalence by 24-h, isolated daytime and isolated nighttime hypertension following ABPM excluding all with WCH. There were 24% (*n* = 18/75) with non-dipping status following ABPM.

**Table 1:** Prevalence of hypertension and phenotypes using 24-h ambulatory BP monitoring or standardised office BP measurement. *

(a) Prevalence of hypertension using ABPM where available (n=75) and office BP only if no ABPM available				
	**Hypertensive**	**Not-hypertensive**		
All, *n* (*%*)	14 (*12*)	102 (*88*)		
< 16, *n* (*%*)	12 (*12*)	90 (*88*)		
≥ 16, *n* (*%*)	2 (*14*)	12 (*86*)		
(b) Blood pressure phenotypes using ABPM (white-coat, sustained and masked hypertension)				
	**WCH**	**Sustained**	**Masked**	**Not-hypertensive**
All, *n* (*%*)	5 (*6*)	2 (*3*)	8 (*11*)	60 *(80)*
< 16, *n* (*%*)	4 (*7*)	2 (*3*)	6 (*10*)	49 (*80*)
≥ 16, *n* (*%*)	1 (*7*)	0 (*0*)	2 (*14*)	11 (*79*)
(c) Hypertension prevalence by 24-h, isolated daytime and isolated nighttime hypertension following ABPM*				
	**Hypertensive**	**Not-hypertensive**		
All, *n* (*%*)	10 *(13*)	65 (87)		
24-h, *n* (*%*)	9 (*12*)			
Isolated daytime, *n* (*%*)	0 (*0*)			
Isolated nighttime, *n* (*%*)	1 (*1*)			

^*^Table 1 (c) hypertensive children excludes *n* = 5 with white coat hypertension

**Fig. 2:**
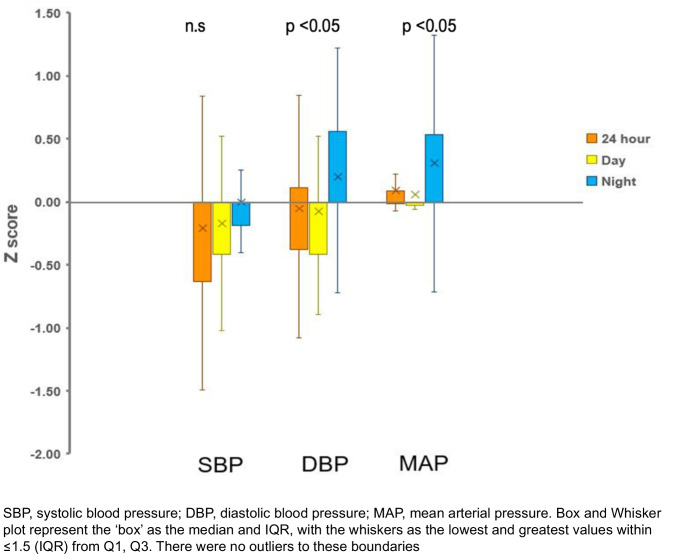
Plot of 24-h, day and night BP *z*-scores for SBP, DBP and MAP for *n* = 75 children who underwent ABPM

Amongst those who were hypertensive, BP values were only mildly elevated with mean office SBP z-score in under 16 s of 1.15 ± 1.2 and DBP 0.7 ± 1.2, equivalent to the 87th and 76th percentile respectively (Supplementary information, Table 1). Figure 2 shows distribution of BP within the cohort, highlighting relatively higher nocturnal BP compared to the reference population.

### Comparison of demographic characteristics between children with ‘High BP’ and those who were normotensive

The prevalence of those with High BP was 26% (n = 30/116). Table 2 compares characteristics of those with High BP with those who were normotensive. Those with High BP were older, but with a similar growth profile compared to those who were normotensive. They had a higher cyst burden but with well-preserved eGFR.

**Table 2:** Demographic and clinical characteristics in 116 untreated children with ADPKD, High BP, ≥ 90th percentile and normotension (<90th percentile). *Mean (SD) unless otherwise stated*

	All (*n* = 116)	Normotension (*n* = 86)	High BP (*n* = 30)	*P*
Age, year,	9.99 (5.23)	9.3 (4.98)	11.3 (5.34)	0.06
Female sex, *n* (%)	67 (57.8)	50 (58.1)	17 (56.7)	0.89
Ethnicity, *n* (%)				
White	72 (62.1)	50 (58.1)	22 (73.3)	
Black	3 (2.6)	2 (2.3)	1 (3.3)	
Asian	15 (12.9)	12 (14.0)	3 (10.1)	
Other	9 (7.8)	7 (8.1)	2 (6.7)	
Not specified	17 (14.7)	15 (17.4)	2 (6.7)	
Height (cm)	136.3 (32.9)	133.9 (31.9)	143.3 (35.2)	0.18
Height *z*-score	0.265 (1.23)	0.29 (1.22)	0.21 (1.23)	0.76
Weight (kg)	38.8 (22.4)	36.9 (21.6)	44.1 (24.1)	0.13
Weight *z*-score	0.323 (1.25)	0.40 (1.21)	0.10 (1.38)	0.26
BMI (kg/m^2^)	18.9 (4.27)	18.8 (4.39)	19.3 (3.95)	0.53
BMI *z*-score	0.284 (1.0)	0.33 (1.05)	0.15 (0.86)	0.41
Creatinine	46.6 (16.9)	44.8 (16.9)	51.4 (16.2)	0.07
eGFR in ml/min per 1.73 m^2^	114.3 (26.2)	117.0 (26.7)	107.3 (24.0)	0.08
Cyst burden (%) *n* = *109*				
0–2, *n* (%)	67 (61.5)	55 (67.1)	12 (44.4)	0.06
3–5, *n* (%)	29 (26.6)	20 (24.4)	9 (33.3)	
6–8, *n* (%)	13 (11.9)	7 (8.5)	6 (22.2)	
Family history ADPKD				0.84
Paternal, *n* (%)	38 (32.8)	29 (33.7)	9 (30.0)	
Maternal, *n* (%)	69 (59.5)	51 (59.3)	18 (60.0)	
Nil known, *n* (%)	9 (7.8)	6 (6.9)	3 (10.0)	
Genetic history				0.91
* PKD1*, *n* (%)	25 (21.6)	19 (22.1)	6 (20.0)	
* PKD2*, *n* (%)	2 (1.7)	2 (2.3)	0 (0.0)	
Unknown, *n* (%)	86 (74.1)	63 (73.3)	23 (76.7)	
Negative, *n* (%)	3 (2.6)	2 (2.3)	1 (3.3)	

### Left ventricular structure, geometry and function

**Fig. 3:**
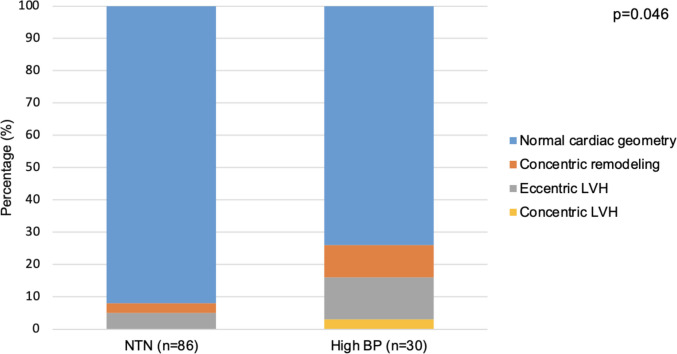
Maladaptive cardiac remodelling in 116 untreated children with ADPKD categorised as High BP (BP ≥ 90th percentile) and normotensive (NTN, BP <90th percentile). LVH, left ventricular hypertrophy

The overall prevalence of LVH was 8% (*n* = 9/116), 16.6% in those with High BP (*n* = 5/30) and 4.6% (*n* = 4/86) in normotensive children with ADPKD (*P* = 0.034). Six children had evidence of concentric remodelling, with a higher prevalence of remodelling in those with High BP when compared with those who were normotensive (8/30 vs. 7/86, *P* = 0.046), respectively (Fig. 3).

**Fig. 4:**
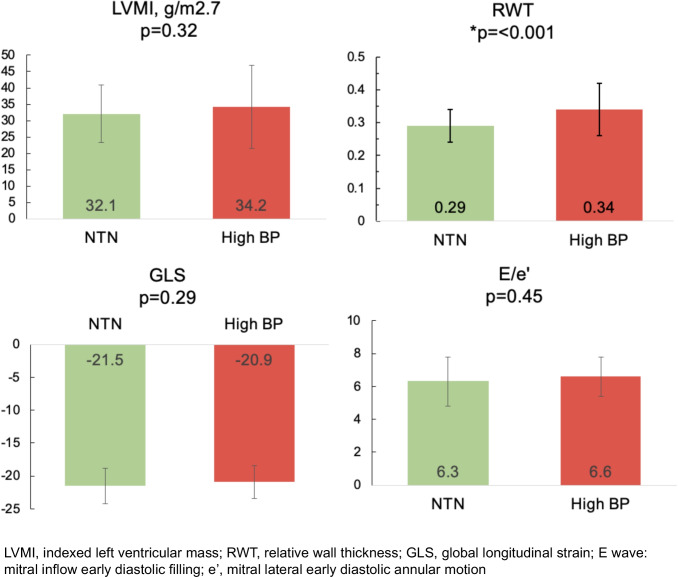
Left ventricular structure and function in 116 untreated children with ADPKD categorised as High BP (BP ≥ 90th percentile) and normotensive (NTN, BP <90th percentile). Error bars represent SD scores

Mean LVMI (g/m^2.7^) was comparable between the two groups, (34.2 ± 12.6 g/m^2.7^ vs. 32.1 ± 8.8 g/m^2.7^, *P* = 0.32); but RWT was significantly higher in those with High BP compared with those who were normotensive (0.34 ± 0.08 vs. 0.29 ± 0.05, *P* < 0.001) respectively. Indexing LV mass in g/m^2^, we observed no significant difference in those with High BP when compared with normotensive children (38.2 ± 8.8 g/m^2^ vs. 41.5 ± 9.0 g/m^2^, *P* = 0.08). There was no difference in LV functional parameters (systolic or diastolic) between those with High BP and those who were normotensive (Fig. 4).

### Characteristics of children with BP ≥ 75th percentile

Those with BP ≥ 75th percentile were older with higher cyst burden, lower eGFR and elevated RWT (Supplementary information, Table 2).

### Determinants of LVMI

Cyst burden showed a significant independent relationship with LVMI g/m^2.7^ (standardised *β*-coefficient = 0.23, *P* = 0.016). Cyst burden was a stronger determinant than office SBP (standardised *β*-coefficient = 0.19, *P* = 0.14). Model adjusted *r*^2^ = 0.334, overall *P*-value < 0.001. Evaluating BP level as a dependent variable, age alone remained as a significant independent predictor (*β* = 0.69, *P* < 0.001) but not cyst score, eGFR or LVMI.

## Discussion

The main findings of our report are that in untreated young children with ADPKD with well-preserved kidney function the prevalence of hypertension was 12% and the prevalence of ‘High BP’ including all those with blood pressure above the ≥ 90th percentile was 26%. Importantly, we observed that in those with High BP there is higher prevalence of LVH and of adverse cardiac remodelling. Finally, as a novel hypothesis generating observation, we highlight higher cyst burden, a marker of more severe ADPKD, to have a significant association with left ventricular mass independent of BP.

Children with ADPKD have CKD by definition. Irrespective of level of kidney dysfunction, lower BP levels in those with childhood CKD has been shown to be of importance for both renoprotection and amelioration of adverse cardiovascular outcomes [28, 29]. The latest KDIGO ADPKD guidelines suggested BP targets to < 75th percentile for childhood ADPKD primarily for renoprotection [22].

Our findings of LVH and adverse cardiac remodelling in those with higher BP, when compared with those with lower BP targets (< 90th and < 75th percentile), underscores the importance of lower BP in this high-risk group. These results provide critical evidence for cardiomyocyte protection and support the recent KDIGO guidelines for children with ADPKD [22].

Hypertension is the main modifiable disease manifestation in children with ADPKD, often preceding a decline in kidney function [30], and a known risk association for worse renal outcomes in young adulthood if it develops under the age of 35 [14, 31]. Despite its relevance, there remain challenges in both the measurement and interpretation of BP in children. The diagnosis of hypertension in children includes percentile definitions for the young, that changes to threshold BP level based definitions in adolescents, with inherent discrepancies that arise through age- or height-related normal values that have been well described elsewhere [20, 32]. The clinical relevance of WCH also remains unclear especially in childhood ADPKD. These issues contrast with adults in whom hypertension definitions relate to cardiovascular risk both in the wider adult population and in those adults with ADPKD [21, 33, 34].

Our observed prevalence of hypertension is at the lower end of the range reported when compared with previous paediatric studies that reported rates of 10–35% [6, 12]. In keeping with previous reports, we observed a similar pattern of higher night time BP resulting in nocturnal non-dipping status [12]. Thus, the predominant phenotype of masked hypertension, and shift towards higher BP at night observed in our study underscores the necessity for vigilant monitoring of BP using appropriate ambulatory methods where possible. In our relatively young population, most patients with hypertension had minimally elevated BP, in keeping with stage 1 hypertension (Supplementary information, Table 1).

The prevalence of LVH in our cohort was low at 8% and, unlike previous studies, we did not see a significant difference in indexed LV mass between those with and without hypertension. We observed an association between High BP and adverse cardiac remodelling, with differences remaining between groups when stratified using the suggested lower clinical target [22]. Our findings are in keeping with those reported in the HOT-KID study, an open-label, randomised control trial (RCT) evaluating cardiovascular outcomes in children with CKD, comparing two different levels of BP, with results supporting lower SBP near the 50th percentile [29]. We accept that differences in growth profiles in children with BP ≥ 75th percentile (compared to those with BP < 75th) may additionally contribute to the observed differences in RWT seen in our study cohort. Alternatively, these differences may reflect the relatively longer duration of higher BP in these older children resulting in the development of the adverse cardiac remodelling. As these data are cross-sectional, we cannot comment on this any further. Overall, our finding of adverse cardiac remodelling despite minimally elevated BP highlights the relevance of comprehensive evaluation for hypertension and its impact on the heart in this population.

In the authors’ clinical practice, we treat all children with ADPKD and hypertension with anti-hypertensive medication preferentially using RAAS inhibitors and with advice regarding non-pharmacological interventions. Currently, we do not treat non-dipping status and target BP below the 75th percentile. In those with adverse cardiac remodelling (including LVH or concentric remodelling) associated with hypertension, we commence anti-hypertensive medication and with advice regarding non-pharmacological interventions, whilst targeting BP < 75th percentile. We re-evaluate management of hypertension more formally with ABPM at 6-monthly intervals and echocardiography in 6–12 months to confirm resolution of LVH. In those with hypertension and concentric remodelling only, we do not routinely repeat echocardiography but manage hypertension with anti-hypertensive medication and non-pharmacological interventions.

The observation of higher cyst burden, independent of BP, to have a significant association with LV mass in our study population merits further discussion. A higher cyst burden may indicate more severely deficient polycystin function resulting in severe cardiac remodelling. Polycystin is known to be expressed in the myocardium, and animal and cell models have suggested that dysfunction of PC1 and PC2 results in hypertrophic remodelling of myocytes [4, 35, 36]. Clinical studies have also previously reported total kidney volume (TKV) to be independently linked to increased LV mass [37]. To our knowledge, clinical data evaluating a potential association of PKD genotype and cardiac remodelling, to test the hypothesis that a polycystin-dependent mechanism is at work, has not been reported previously in children with ADPKD. Recent work in adults highlighted a significant association between LVH and *PKD1* mutations, independent of hypertension and other confounders, suggesting that a polycystin-dependent mechanism may be at play [38]. Taken together, these findings raise the intriguing idea that BP-independent, and polycystin-dependent mechanisms may be involved in the development of cardiac remodelling even at these very early stages of ADPKD and merit further clinical and basic science research.

We recognise additionally the importance of identifying other modifiable risk factors common to all patients with a degree of CKD, when assessing children with ADPKD. These include targeting increased BMI, reducing dietary intake of excess sodium, reducing exposure to nephrotoxins and avoiding smoking [22, 39].

Limitations are recognised and inherent in a single centre, retrospective study design like ours. Despite this we have included all consecutive children eligible for inclusion with comprehensive assessment of their BP characteristics, kidney imaging and performed detailed echocardiography. We did not measure cystatin C concentrations and estimated kidney function using the new bedside Schwartz formula [16, 17]. We additionally recalculated the eGFR using the FAS (Full Age Spectrum) equation for height and using plasma creatinine as it has been suggested that the FAS equation performs better across transitional ages, reduces bias related to height extremes and tends to perform better at higher GFR ranges, especially those with near-normal kidney function such as our study population [40, 41]. There were though no differences to our study conclusions when substituting eGFR calculated using Schwartz with eGFR by FAS and we have therefore shown results using the eGFR by the bedside Schwartz formula only. We accept that the ideal indexation of measured LV mass using echocardiography remains debatable with different methods in the published literature although we did not find any change in our study findings using different indexation method. Our study report is also in keeping with methods reported from the only RCT evaluating cardiovascular outcomes in children with CKD and definitions of LVH using widely accepted age range-based reference intervals [23]. Our study adds to the relatively sparse information regarding BP and association on the cardiovascular aspects in childhood ADPKD, with ~ 300 children included over previously published studies. Collaboration between multiple centres and potential use of registry data would help to further mitigate these limitations. Genetic evaluation was only available for 25% of patients included in our study cohort, and in keeping with gene testing not currently clinical standard in the UK. It could be theorised that most children will have *PKD1* mutations given childhood presentation – however confirmatory genotyping would help draw conclusions of relationship between this and phenotypic findings. More precise assessment of cyst burden using magnetic resonance imaging (MRI) to calculate total kidney volume (TKV) would be ideal but remain limited in childhood clinical cohorts such as ours [42]. Prospective longitudinal studies are also needed to establish optimal BP thresholds and their effect on cardiovascular and renal outcomes in childhood ADPKD similar to those that have been shown in adults with ADPKD [43, 44].

## Conclusion

In this group of untreated children with ADPKD and preserved kidney function, there is a clinically significant prevalence of hypertension including masked hypertension, with High BP associated with LVH and adverse cardiac remodelling. The independent association of cyst burden and adverse cardiac remodelling is hypothesis generating, suggesting a BP-independent role of polycystin in its pathogenesis. Further research is needed to investigate these associations and continue to elucidate the underlying mechanisms for LV remodelling in this cohort.

## Supplementary Information

Below is the link to the electronic supplementary material.

Graphical abstract (TIF 175 KB)

Supplementary material 1 (DOCX 26.7 KB)

## Data Availability

The datasets generated during and/or analysed during the current study are available from the corresponding author on reasonable request.
