# Supplementary material for: Cystic burden meets blood pressure: cardiac remodelling in paediatric autosomal dominant polycystic kidney disease
Source: Pediatr Nephrol. 2026 May 18;41(10):3477–87. doi: 10.1007/s00467-026-07312-8 (PMC13481587; doi:10.1007/s00467-026-07312-8)
Supplement: Supplementary file 1 [file 467_2026_7312_MOESM2_ESM.docx]

**Supplementary Information**

**Article Title:** Cystic Burden Meets Blood Pressure: Cardiac Remodelling in Paediatric Autosomal Dominant Polycystic Kidney Disease

**Journal Name:** Paediatric Nephrology

**Authors**: Camille Heming^1^, Emily Haseler^2^, Alex Savis^3^, Hannah Samuel^1^, John M Simpson^2,3^, Manish D. Sinha^1,2*^

**Affiliations**^: 1^Department of Paediatric Nephrology, Evelina London Children’s Hospital, Guys & St Thomas NHS Foundation Trust, Westminster Bridge Road, London SE1 7EH; ^2^Department of Clinical Pharmacology, School of Cardiovascular and Metabolic Medicine, King’s College London; ^3^Department of Paediatric Cardiology, Evelina London Children’s Hospital, Guys & St Thomas NHS Foundation Trust, Westminster Bridge Road, London SE1 7EH

^*^**Corresponding author**: Professor Manish D Sinha, Department of Paediatric Nephrology, 3^rd^ Floor Beckett House, Evelina London Children’s Hospital, Guys & St Thomas NHS Foundation Trust, Westminster Bridge Road, LONDON SE1 7EH, United Kingdom.

**Supplementary Table 1:** Office and Ambulatory BP characteristics of whole cohort, normotensive and hypertensive patients. *P value pertains to normotensive compared to hypertensive group*.

|  | All  (n=116) | Normotensive  (n = 102) | Hypertensive  (n=14) | p-value |
| --- | --- | --- | --- | --- |
| Office SBP mmHg | 104±14 | 102.4 ± 12.4 | 118.6 ± 15.6 | <0.001 |
| Office SBP z score (<16, n=102) | 0.16±0.8 | -0.02 ± 0.74 | 1.15 ± 1.2 | <0.001 |
|  |  |  |  |  |
| Office DBP mmHg | 64±10 | 63.1±9.17 | 71.6±12.3 | 0.004 |
| Office DBP z score (<16, n=102) | 0.16±1.1 | 0.09 ±1.1 | 0.7±1.3 | 0.09 |
|  |  |  |  |  |
| ABPM (n=75)  **z scores by age* | **All**  **(n=75)** | **Normotensive**  **(n=65)** | **Hypertensive**  **(n=10)** | p-value |
| 24h MAP mmHg | 81±6 | 79.8±5.1 | 91.7±5.7 | <0.001 |
| 24h MAP z score* (<16, n=61) | 0.12±1.1 | -0.15±0.82 | 2.0±1.04 | <0.001 |
| 24h SBP mmHg | 109±9 | 106.9±7.6 | 122.4±6.9 | <0.001 |
| 24h SBP z score* (<16, n=61) | -0.40±1.3 | -0.68±1.06 | 1.49±1.16 | <0.001 |
| 24h DBP mmHg | 66±7 | 64.4±5.3 | 76.8±6.1 | <0.001 |
| 24h DBP z score* (<16, n=61) | -0.14±1.2 | -0.41±0.92 | 1.68±1.07 | <0.001 |

**Supplementary Table 2**: Demographic and clinical characteristics in 116 untreated children with ADPKD, stratified by standardised office BP < 75^th^ percentile, and ≥75^th^ percentile

|  |  | BP <75^th^ percentile  (n=60) | BP≥75^th^ percentile (n=56) | | p |
| --- | --- | --- | --- | --- | --- |
| Age, yr,  *mean (SD)* |  | 8.34 (5) | | 11.4 (4.9) | <0.001 |
| Height (cm) |  | 127.2 (32.6) | 146.1(30.7) | | 0.002 |
| Height z-score *mean (SD)* |  | 0.15 (1.3) | 0.39 (1.1) | | <0.001 |
| Weight z-score *mean (SD)* |  | 0.17 (1.02) | 0.48 (1.5) | | <0.001 |
| BMI z-score *mean (SD)* |  | 0.23 (0.97) | 0.34 (1.04) | | <0.001 |
| Creatinine *mean (SD)* |  | 40.5 (148) | 53 (16.8) | | <0.001 |
| eGFR in ml/min per 1.73m^2^  *mean (SD)* |  | 121.7 (27.1) | 106.7 (23.1) | | <0.001 |
| “High” cyst burden n (%) |  | 16 (27) | 26 (53) | | 0.005 |
|  |  |  |  | |  |
| LV Structure |  |  |  | |  |
| RWT (mean±SD) |  | 0.28±0.05 | 0.32±0.07 | | **<0.001** |
| LVMI, g/m^2.7^, (mean±SD) |  | 32.5±9.3 | 33.5±10.9 | | 0.904 |
| LV Function |  |  |  | |  |
| LV E/e' (mean±SD) |  | 6.6±1.6 | 6.2±1.1 | | 0.22 |
| GLS, -ve, (mean±SD) |  | 21.5±2.8 | 21.2±2.5 | | 0.49 |
| EF% (mean±SD) |  | 64.5±4.6 | 63.9±6.2 | | 0.69 |
